# Smartphone-based photogrammetry provides improved localization and registration of scalp-mounted neuroimaging sensors

**DOI:** 10.1038/s41598-022-14458-6

**Published:** 2022-06-27

**Authors:** Ilaria Mazzonetto, Marco Castellaro, Robert J. Cooper, Sabrina Brigadoi

**Affiliations:** 1 Department of Developmental and Social Psychology, University of Padova, via Venezia 8, 35131 Padua, Italy; 2 Department of Information Engineering, University of Padova, via Gradenigo 6/b, 35131 Padua, Italy; 3 Department of Neuroscience, Biomedicine and Movements, University of Verona, P.le L.A. Scuro 3/A, Verona, Italy; 4 DOT-HUB, Biomedical Optics Research Laboratory, Department of Medical Physics and Biomedical Engineering, University College London, London, WC1E 6BT UK

**Keywords:** Biomedical engineering, Near-infrared spectroscopy, Neuroscience

## Abstract

Functional near infrared spectroscopy and electroencephalography are non-invasive techniques that rely on sensors placed over the scalp. The spatial localization of the measured brain activity requires the precise individuation of sensor positions and, when individual anatomical information is not available, the accurate registration of these sensor positions to a head atlas. Both these issues could be successfully addressed using a photogrammetry-based method. In this study we demonstrate that sensor positions can be accurately detected from a video recorded with a smartphone, with a median localization error of 0.7 mm, comparable if not lower, to that of conventional approaches. Furthermore, we demonstrate that the additional information of the shape of the participant’s head can be further exploited to improve the registration of the sensor’s positions to a head atlas, reducing the median sensor localization error of 31% compared to the standard registration approach.

## Introduction

Electroencephalography (EEG) and functional near-infrared spectroscopy (fNIRS) are two non-invasive techniques that can measure the neural/hemodynamic activity of the brain. EEG and fNIRS data are measured from the head surface by placing several sensors (from a few to hundreds) over the scalp of the subject.

fNIRS measures changes in the intensity of light emitted by a source on the scalp and backscattered to a detector placed nearby to recover concentration changes of oxy (HbO) and deoxy-hemoglobin (HbR) occurring in the superficial cortex^1^. Each source and detector placed close enough to one another such that a measurement can be made is considered a “channel” To localize the measured activity within the cortex, one of the most common and straightforward approach consists in projecting the central point of the channel onto the cortical surface and identifying either the coordinates in the atlas space or the brain region^2^. This approach assumes that the centre of an fNIRS channel corresponds to the cortical location to which that channel is most sensitive, which is not necessarily the case in a complex geometry like the human head. A more advanced approach consists in computing the cortical sensitivity of the channels based on models of photon propagation through the human head^3,4^. Whatever approach is employed, an accurate knowledge of the location of sensors on the individual’s scalp is fundamental to determine the cortical location of the measured activity and to evaluate the variability in probe placement across subjects and acquisitions^5^. While the spatial resolution of sparsely distributed single-distance fNIRS arrays is approximately 30 mm^6^, the spatial resolution of high-density arrays can reach approximately 13 mm^6,7^. This further highlights the importance of accurately detecting sensor locations on individual participants.

EEG measures the electrical activity produced by the postsynaptic potentials of neuronal populations. Since this activity is generated several centimetres below the scalp, it travels through different resistive layers before being measured at the surface^8^. These layers, especially the skull^9^, cause a blurring effect at the outer layer. Therefore, the recorded activities are weighted sums of the underlying brain sources. For this reason, in contrast to fNIRS, the cortical activity cannot be localized by simply projecting the sensor positions from the scalp to the cortex. To identify the brain sources, a head model, describing the geometrical and electrical properties of the tissues through which the currents flow and a model describing how the scalp potentials result from the current dipoles inside the head are required. The accuracy of the source estimation strongly depends on the degree of approximation adopted to describe the head model^10^. Furthermore, the precision of sensor positions has been shown to be very important. Computational studies using a realistic head model reported that an error of 5 mm (or a rotation of 5° backwards or to the left) in sensor positions resulted in a displacement of 2–12 mm in the estimated source locations^11^.

To obtain reproducible results, sensor positions, with both fNIRS and EEG, are usually specified according to the 10-20 system^12^ or its extensions, the 10-10 and 10-5 systems^13,14^. These systems define a set of scalp positions based on the distances between cranial landmarks (i.e., nasion, inion, left and right preauricular points), so that positions are consistent across different head sizes. The assumption is that there exists a correspondence between a point on the head surface and the underlying cortical region^15^. In most studies, EEG/fNIRS sensors are mounted on elastic caps that can be stretched to fit the head of the participant. The inter-sensor distance can therefore vary from subject to subject. The caps are usually available in different sizes and are usually positioned on the participant’s head by centring the cap over the apex position Cz. Even if a fixed relative placement is used, scalp and cortical shapes vary across subjects and will not perfectly scale with head circumference, implying that the same channel might be in slightly different locations across subjects. Furthermore, the process of landmarks identification, required to correctly fit the cap, can be ambiguous and prone to subjective errors^16^.

Conventional approaches for the localization of EEG/fNIRS sensors include electromagnetic^17,18^ and ultrasound digitizers^19,20^. Both techniques require the sensor to be touched with a stylus to detect its position. Since positions are registered one at a time, these methods are time consuming for both the operator and the participant, especially with high-density configurations. As a further drawback, the digitization process is user-dependent, error-prone, very sensitive to environmental conditions and very expensive^21^.

Alternative approaches use photogrammetry^22–28^ or 3D scanning^29–33^ methods. Photogrammetry consists of the acquisition of multiple pictures of the subject’s head from different angles while the subject is wearing the cap. Sensors are then manually or automatically identified in each picture and their positions are computed^22–25,34^. Alternatively, pictures can be converted in a 3D point cloud from which the sensor positions as well as the head surface can be derived^26–28^. The 3D scanning technique is similar. In this case, the 3D point cloud is directly generated from acquisitions with either structured light, infrared structured light, or lasers. Overall, sensors localization from a 3D point cloud has been shown to be more accurate, easy to operate, reliable and less sensitive to human error compared to the electromagnetic digitization technique. Furthermore, photogrammetry/3D scanning-based approaches considerably reduce the acquisition time and require minimal manual intervention during the analysis if combined with automated methods for sensor identifications^26,27,32,33^. Compared to electromagnetic and ultrasound digitizers, the time required to post-process the data is, however, longer. Recently, it has been demonstrated that these techniques can improve the EEG source model accuracy^29^.

Whatever approach is employed to localize sensor positions, the outcome is a set of sensor positions and cranial landmarks in an arbitrary space. These positions have therefore to be realigned to the participant’s head or to a generic head model based on an MRI atlas. When a structural MR image of the participant is available, a combination of a transformation mapping the cranial landmarks defined in the sensors space to the cranial landmarks in the MR space, and the iterative closest point (ICP) algorithm^35^, are commonly used. The former is usually performed by estimating an affine transformation: a linear registration with 12 degrees of freedom (translation, rotation, skew and scaling). In most fNIRS and EEG studies, however, the MRI of the participant is not available. In those cases, sensor positions can be mapped to an atlas which can be used in place of the individual MRI^36–38^. There are several ways this mapping procedure can be performed. One straightforward approach is to apply an affine transformation between landmark positions of the individual subject in the sensor space and those of the atlas^27,28,39–41^. An alternative approach consists of defining a set of points based on the extended 10-20 system in both spaces and use a non-linear transformation for the mapping^36^. Since the head has an ellipsoidal shape, there are several possible alignments of the convex hull, defined by the sensor positions, with the atlas head shape. Thus, a registration technique based only on landmarks and/or a few sensor positions may not always lead to accurate results.

An incorrect or inaccurate registration of the individual sensor positions to the atlas may frustrate the improved accuracy in sensor localization achieved with the photogrammetry/3D scanning -based methods. Moreover, with the advent of photogrammetry/3D scanning -based methods, additional information relative to head shape and size are recorded compared to electromagnetic digitization approaches. This opens up the possibility to exploit this supplementary information to accurately register sensor positions to an atlas or to register the atlas to the subject’s head shape. The 3D head model reconstruction from smartphone-based photogrammetry approaches has been already successfully implemented in fields requiring very high level of precision and accuracy, i.e., the analysis of cranial deformation and the definition of a digital facial impressions for maxillofacial prosthesis^42,43^.

In this study, we introduce a photogrammetry-based method for sensor localization that does not require any specific instrumentation, but a smartphone. First, we validate the proposed approach against a typical electromagnetic digitization technique. Second, we investigate which is the best approach to register the obtained sensor positions to an atlas, by testing three different methods: (1) an affine transformation between landmarks (the standard approach); (2) the point-set registration method^44^ between the nodes of the individual 3D model and the nodes of the atlas mesh (applying either linear or non linear transformation), and (3) a volume-based registration method^45,46^ from a 3D image of the individual scalp obtained from the 3D model, to a 3D image of the atlas scalp (applying either linear or non linear transformation).

## Materials and methods

### Validation study

#### Phantom creation

To evaluate the performance of photogrammetry as compared to electromagnetic digitization, and therefore to validate the method, we 3D printed at full scale with an Ultimaker 2+ (Ultimaker B.V., Netherlands) a head phantom on which the positions of the 10-5 EEG system^14^ and of nasion, inion, and left/right preauricular points were marked as 3 mm diameter hollows. The scalp surface mesh, based on the MNI 152 template^47^ and available via www.ucl.ac.uk/DOT-HUB, was created as described in Brigadoi et al. ^48^.

#### Detection of sensor positions

Sensor positions were detected from the 3D phantom with both a photogrammetry-based method and an electromagnetic digitizer (Patriot Polhemus, Colchester, VT).

##### Photogrammetry

Since photogrammetry relies on colour identification, sensor positions were highlighted from the scalp by filling the hollows with modelling clay of different colours based on their positions. The acquisition consisted of a video capture of the entire head phantom.

**Figure 1:**
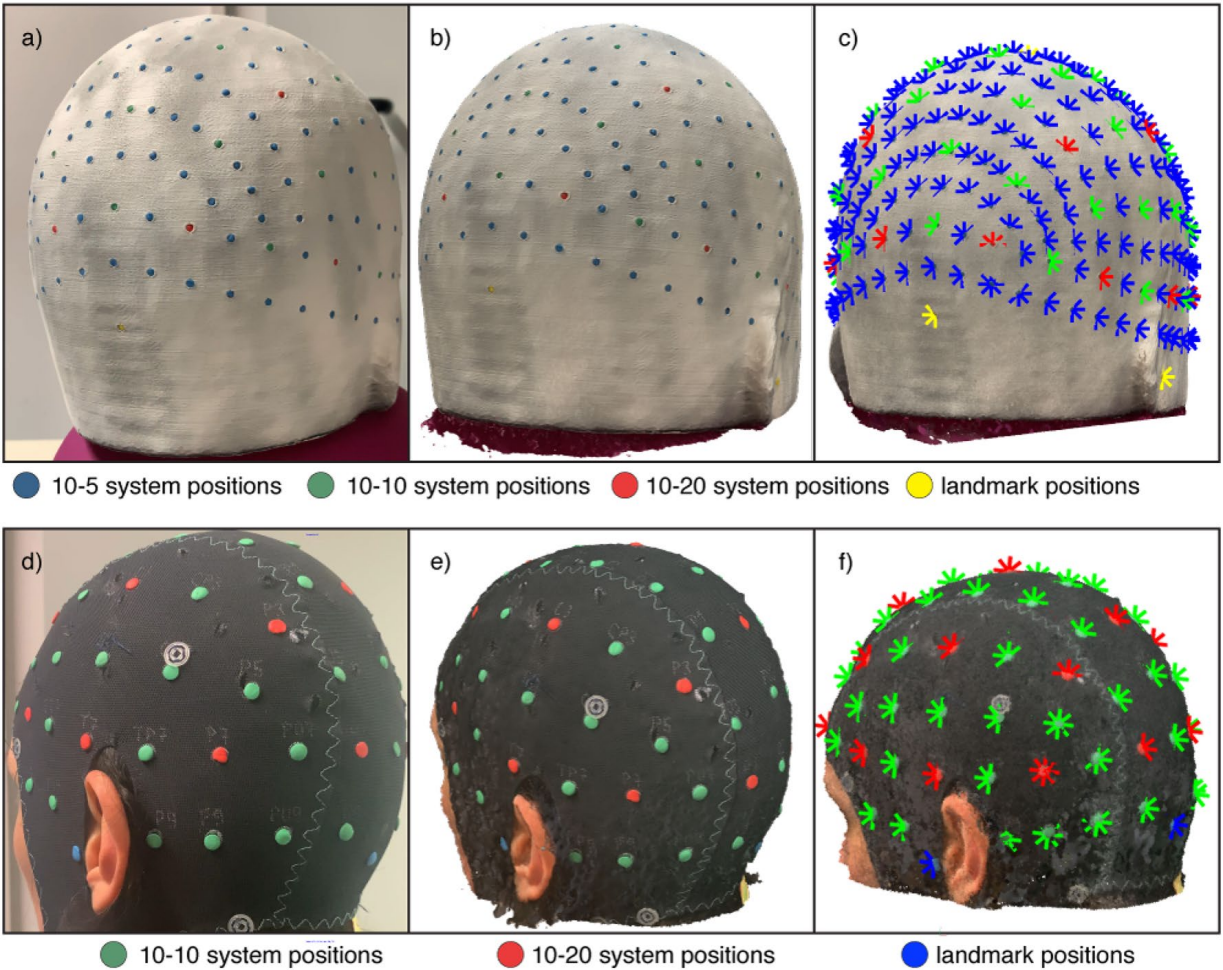
(**a**) Exemplary frame extracted from the recorded video of the phantom marked with the positions of the 10–5 EEG system. (**b**) Reconstructed 3D model of the head phantom obtained with the photogrammetry-based method. (**c**) Locations of the markers identified on the mesh of the phantom. (**d**) Exemplary frame extracted from the recorded video of one of the participants marked with the positions of the 10-10 EEG system and of the main landmarks, (**e**) Reconstructed 3D model of the participant’s head obtained with the photogrammetry-based method. (**f**) Locations of the markers identified on the mesh.

During the video acquisition, the phantom was placed in a normally lit room on a turning support that was slowly turned around three times by an experimenter. Another experimenter stood in front of the phantom and kept the smartphone approximately 40 cm away so that the phantom occupied most of the field of view. The smartphone was kept at the phantom’s eye-level and perpendicular to the horizontal plane during the first lap and then lifted and tilted forward of around 10 cm and 20 degrees each lap. This procedure assured that the video wholly captured the phantom, from the neck to the top. To evaluate the sensitivity of the photogrammetry-based method to camera features, videos were acquired with four different cameras, with camera resolutions and frame rates as follows: Apple iPhone Xs (3840 × 2160 pixels, 60 fps), Asus Zenfone Max Pro M1 (1920 × 1080 pixels, 30 fps), Samsung Galaxy A7 2018 (1920 × 1080, 30 fps with optical image stabilization) and OnePlus 2 (3840 × 2160 pixels, 30 fps). To create a subset of images to be used for 3D model generation, from each video, one frame every 1.3 s was selected. Figure 1a shows an exemplary frame.

The 3D model was built using Agisoft Metashape Standard Edition, version 1.5 (2019). This software represents a cheap solution (educational license costs around 60 US dollars) to obtain the mesh of an object starting from a sample of its images. An exemplary mesh from which the sensors were identified is showed in Fig. 1b. Each mesh was imported into Matlab R2018b (Mathworks, MA, USA) and colour information linked to each node was converted from RGB (Red, Green, Blue) to HSV (Hue, Saturation, Value) scale^32,49^. To easily isolate the nodes representing the sensor positions from the background, a specific range of values was manually defined for the HSV scale for each colour to be identified. All the identified points were then clustered based on their Cartesian coordinates. Nodes distanced less than 3 mm (the diameter of the marker) were assumed to belong to the same cluster. Lastly, the Cartesian coordinates of the sensor positions were estimated as the centre of mass of the different clusters (Fig. 1c).

The quality of each reconstructed mesh was assessed following the procedure described in Clausner et al.^26^. The reconstructed mesh was roughly aligned to the original mesh of the phantom using an affine transformation based on landmarks. Then, the registration to the original mesh was refined by applying the iterative closest point (ICP) algorithm^35^. The accuracy of each node of the reconstructed mesh was computed as the Euclidean distance between that node and the closest node in the original mesh.

##### Electromagnetic digitization

We used the Patriot Polhemus with one transmitter, fixed to the plane where the phantom was placed, a receiver (a stylus pen) and a system attached to a semi-circular plastic support that was fixed on the phantom. This electromagnetic acquisition was performed before inserting the modelling clay in the phantom’s holes. To evaluate the sensitivity of the electromagnetic digitizer to inter-subject variability in the detection of sensor/landmark positions, four different researchers performed the digitization task of all positions. The output of the electromagnetic digitization consists in 3D coordinates (x, y and z) of the digitized points. The static accuracy position of the device, as reported by the manufacturer, is 1.52 mm.

#### Localization performance evaluation

Using some of the functions of the AtlasViewer package (github.com/BUNPC/AtlasViewer) the sensor positions, acquired with each technique, were mapped to the reference system of the head template by computing an affine transformation from the acquired landmark positions to their true position on the phantom and applying this transformation to all acquired sensor positions.

For each sensor, localization error was defined as the Euclidean distance between its estimated position and the true position on the head template.

### Registration study

#### Dataset

In-vivo data were acquired in six participants (four women and two men, age range: 25–35 years) who already had available their own 3D T1 weighted (TIw) MRI. Before taking part to the experiment, participants gave their written informed consent to provide their anatomical MRI to the experimenters and to be recorded with a camera. MRIs of each participant were acquired at 3T with an isotropic resolution of 1 × 1 × 1mm and used in other studies. The study was approved by the Ethics Committee of the Psychological Research Areas of the University of Padova, Italy (protocol number 4362). The in-vivo data were acquired in accordance with the Declaration of Helsinki.

Videos were collected while participants wore a black elastic cap (EASYCAP, Brain Products GmbH) on which the manufacturer had marked the sensor layout based on the 10–10 system. To make those positions identifiable with photogrammetry, a 5 mm diameter, 1 mm thick modelling clay disk was placed over each labelled position (Fig. 1d). During a real EEG/fNIRS experiment, this situation could be reproduced adding coloured circular stickers on top of the sensors. Video recording and head mesh generation were performed following the procedure described in the validation study. Regions of no interest (e.g., the neck) were cut using Meshlab^50^. An example of a participant’s head mesh from which the sensor positions were identified is shown in Fig. 1e.

Landmark and sensor positions were identified automatically as described in the validation study and the detected positions were then projected to the nearest node of the scalp mesh (Fig. 1f).

#### Benchmarks definition

In order to evaluate the performance of each registration method to a generic atlas, two different benchmarks were evaluated.

The definition of the benchmarks requires some pre-processing steps of the individual MR images. Briefly, MR images were segmented by computing the probability maps of grey matter, white matter, cerebrospinal fluid, skull, skin and air using the “unified segmentation” algorithm^51^ implemented within SPM12 (http://www.fil.ion.ucl.ac.uk/spm/software/spm12/). A multi-layered tissue mask was obtained assigning each voxel to the tissue class with the highest probability. Using this mask, a multi-layered volumetric mesh was created using the iso2mesh toolbox^52^, with the CGAL mesher option (http://www.cgal.org). The outward layer of the volumetric mesh was isolated and used as scalp surface.

The first benchmark was defined as the positions of the sensors on the scalp of the head template as mapped with a brain-to-brain registration between individual and the atlas model MRIs. First, sensor positions derived from photogrammetry were mapped to the individual MR space. This step was carried out by aligning the individual mesh obtained from the photogrammetry approach to the individual scalp surface derived from the subject’s structural MRI image. Before the realignment, to ease the computational burden, the scalp meshes were downsampled using a 3D box grid filter in order to obtain a number of nodes around 3000. Alignment was performed in two steps, the first to orient the two meshes in the same direction and the second performing the actual alignment. For the first step, three points were manually chosen in locations around the two ears and the nose in both surfaces and used to estimate a rigid transformation that roughly aligned the two meshes in the same space and direction. For the second step, an affine registration was computed using the Coherent Point Drift algorithm (CPD)^44^ implemented in the Matlab package CPD (http://www.bme.ogi.edu/~myron/matlab/cpd/). Sensor positions in the individual MR space were obtained by applying the rigid and affine transformations to the sensor positions derived from the photogrammetry model. Sensor positions in the individual MR space were then mapped to the template by applying a brain-to-brain transformation that warped the individual structural brain image to the brain template. Individual brain image was extracted from the structural image using Multi Atlas Skull Stripping (MASS) software (https://www.nitrc.org/projects/cbica_mass/) after a preliminary step aiming to correct for the low frequency intensity inhomogeneity (bias field) with N4 algorithm^53^. The transformation was computed with the software Advanced Normalization Tools (ANTs)^45^ and consisted of a combination of a linear registration using mutual information as similarity metric and a non-linear registration using the Symmetric Normalization algorithm in combination with cross correlation^54^. Each sensor position, defined at the voxel level in the atlas space, was then projected to the nearest node of the atlas scalp surface.

The second benchmark consisted in determining the cortical location with the highest fluence distribution for each sensor in the head template. The photon fluence associated with each sensor was simulated in the individual multi-layered volumetric mesh with a Monte Carlo approach using the MCX package^3^. Each sensor was considered as a source, which was modelled as a pencil beam, and the number of simulated photons was set to 10^9^. Optical properties were assigned based on literature^55–57^. The fluence distribution of each sensor was then mapped to the atlas model by applying the same brain-to-brain transformation described above. For each sensor, fluence distribution was mapped to the grey matter surface of the head model template as described in Brigadoi et al.^48^. For each sensor, the cortical location with the highest fluence was identified by selecting all nodes exceeding 80% of the maximum value of the fluence distribution and averaging their spatial coordinates weighted by their fluence values.

The process of defining these benchmarks yielded, for each sensor position, a ‘true’ coordinate on the atlas scalp surface and a ‘true’ cortical location associated with peak optical fluence.

#### Registration approaches

Sensor positions measured with the photogrammetry approach were mapped to the head template using five different transformations estimated with the following approaches.

##### Affine registration with landmarks

The affine transformation was estimated using the least square method from nasion, inion, Cz, left and right preauricular points defined in the individual and template scalp surface.

##### CPD with an affine registration

The individual and template scalp surfaces were downsampled using a 3D box grid filter in order to obtain a number of nodes around 3000. To obtain a surface smoothness comparable to the one of the template, the photogrammetry-derived mesh was smoothed with a low pass filter, which was shown by Bade et al.^58^ to be the best volume preserving smoothing algorithms. Surfaces were first aligned by applying a simple rigid transformation, estimated using four easily detectable points on a mesh, i.e., nasion, Cz, left and right preauricular points. Then, both individual and template scalp surfaces were cut with an axial plane under the nasion. This pre-processing step was required to avoid the registration to be biased by differences in the slope of the nose between the two surfaces. Finally, individual and atlas scalp surfaces were aligned using the Coherent Point Drift algorithm (CPD)^44^.

##### CPD with a non-linear registration

The same procedure previously described for the affine registration was applied here, using a non-linear registration instead of the affine one. The only different pre-processing step was the downsampling one, which retained approximately 9000 nodes, providing a trade-off between computational burden and preservation of an acceptable resolution for sensor positions. The non-linear registration problem is defined as an initial position plus a displacement function. To force close points to move coherently, the displacement function has to be smooth and this can be achieved by regularizing its norm^44^. Three parameters have to be set for the regularization: ω, the amount of noise in the point set, λ, the model of the smoothness regularizer, and β, the trade-off between the goodness of fit and regularization. Based on several tests performed to optimize the function to our data, parameters were set equal to 0.4, 5, and 4.

##### ANTs with an affine registration

As in the CPD-based registrations, the individual surface was downsampled, smoothed, rigidly aligned with the template and cut under the nose. Individual surfaces were then converted to 3D NIfTI images^59^, with a grid step for the resulting volume equal to 1.5 mm. Another 3D NIfTI image of the same size was created, containing four 3 mm radius spheres located in correspondence of the positions of four of the cranial landmarks (nasion, Cz, left and right preauricular points). The same procedure was applied to the template scalp surface and landmarks. Affine mapping between individual and template was computed with ANTs as the sum of a rigid and an affine transformation. Rigid and affine transformations were estimated from the landmark images and surface images, respectively. In both steps, the Point-Set Expectation was employed as metric^60^. The standard deviation of the Parzen window, used to estimate the expectation, and the number of neighbours, used to compute the deformation, were set equal to 600 and 5, respectively.

##### ANTs with a non-linear registration

The same procedure previously described for the affine transformation was applied here, but replacing the affine transformation with a non-linear transformation, using the Symmetric Normalization algorithm, with the Point-Set Expectation as metric^60^.

The CPD algorithms were run using a regular personal computer equipped with a 3.00 GHz quad core 64*-bit Intel(R) Core™ i5-7400* processor with 16 GB of RAM. Linear and non-linear registrations with ANTs were instead computed on a node of a high performance computing cluster server equipped with 8 processors 12-Core Intel Xeon Gold 5118 at 2.30 GHz and 1.5 TB RAM.

For each registration method, photon fluence was re-computed for each registered sensor in the atlas space using the same procedure described for the benchmark definition.

**Figure 2:**
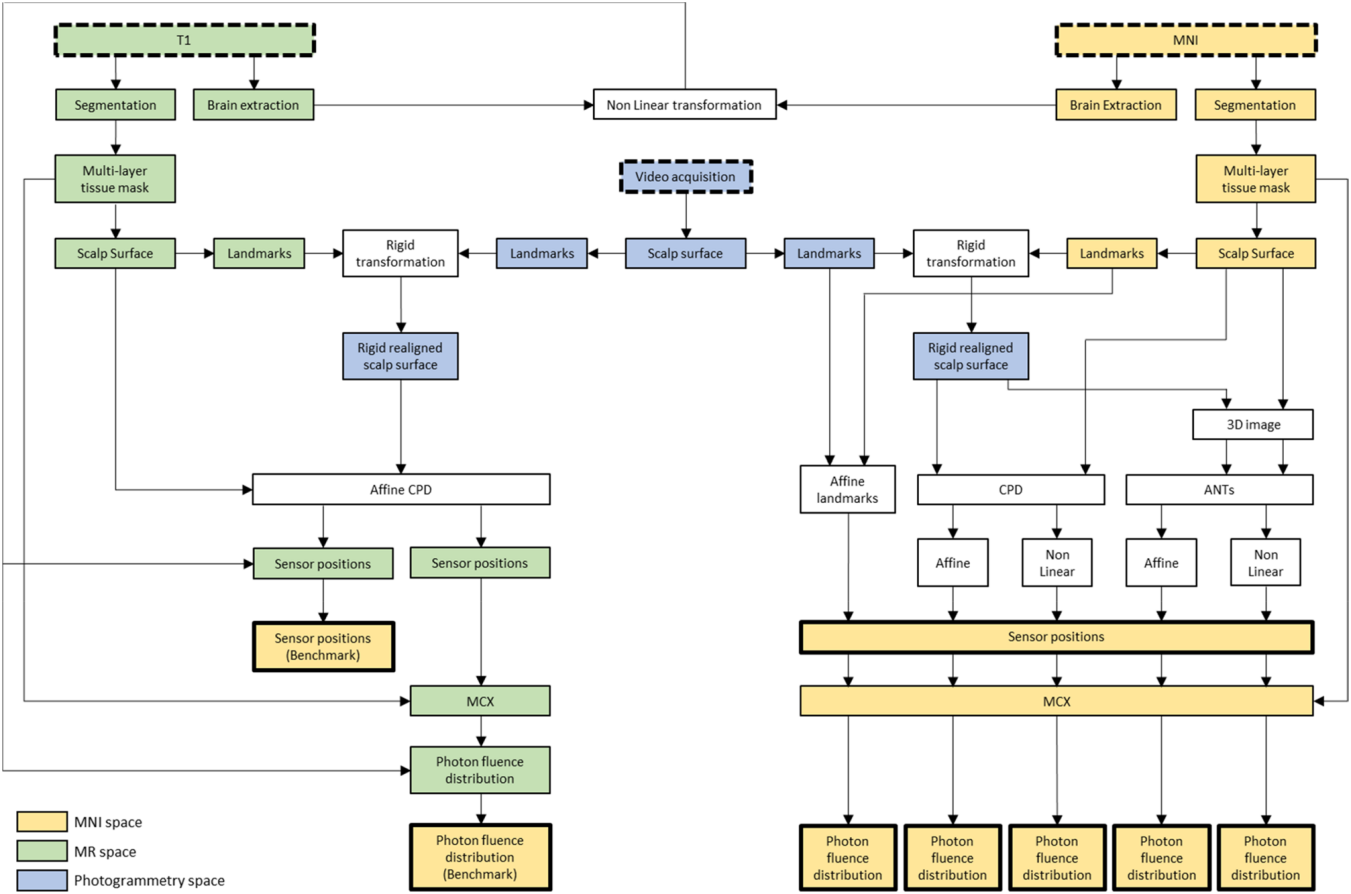
Schematic of the workflow of the registration study. Dashed boxes highlight the data required to perform the analyses, whereas the thicker boxes identify the final outputs, which were then compared to assess the best approach to map the sensor positions from the individual surface to the atlas template surface.

A schematic overview of the registration study is reported in Fig. 2.

#### Registration performance evaluation

To determine the registration approach yielding the lowest error, for each sensor and registration method, the Euclidean distance between the benchmarks and the registered sensor positions/fluence distributions were computed. In order to assess the spatial overlap between the benchmark and the photogrammetry-based fluence distributions, the Dice similarity was also evaluated. This metric was computed considering the regions composed of all nodes with a value greater than 80% of the maximum value of the fluence distributions. The higher the dice coefficient, the higher the overlap between the two regions.

For each metric, the errors from all participants and sensors were compared across the five registration approaches by means of paired two-sided Wilcoxon rank tests corrected for multiple comparisons (*p* < 0.05) with the FDR approach^61^.

## Results

### Validation study

The time required to digitize the 254 marker positions on the head phantom using Polhemus was on average 9.8 min (range 7.3–12.8 min). By contrast, the videos were recorded, on average, in 2.9 min (range 2.5–3.5 min).

The photogrammetry-based method required, however, some post-processing steps. This additional computational time was mainly due to the mesh generation process and depended on the number of frames employed in the reconstruction and the desired accuracy. On average, the total time required to create the mesh was 119.5 min (range 65.1–170.8 min).

The number of nodes of the meshes was strongly related to the smartphone resolution: meshes derived from smartphones with higher resolution (3840 × 2160 pixels) had, on average (SD), 1,610,780 (412,136) nodes, whereas the meshes derived from the smartphones with lower resolution (1920 × 1080 pixels) had, on average (SD), 468,461 (22,780) nodes.

**Figure 3:**
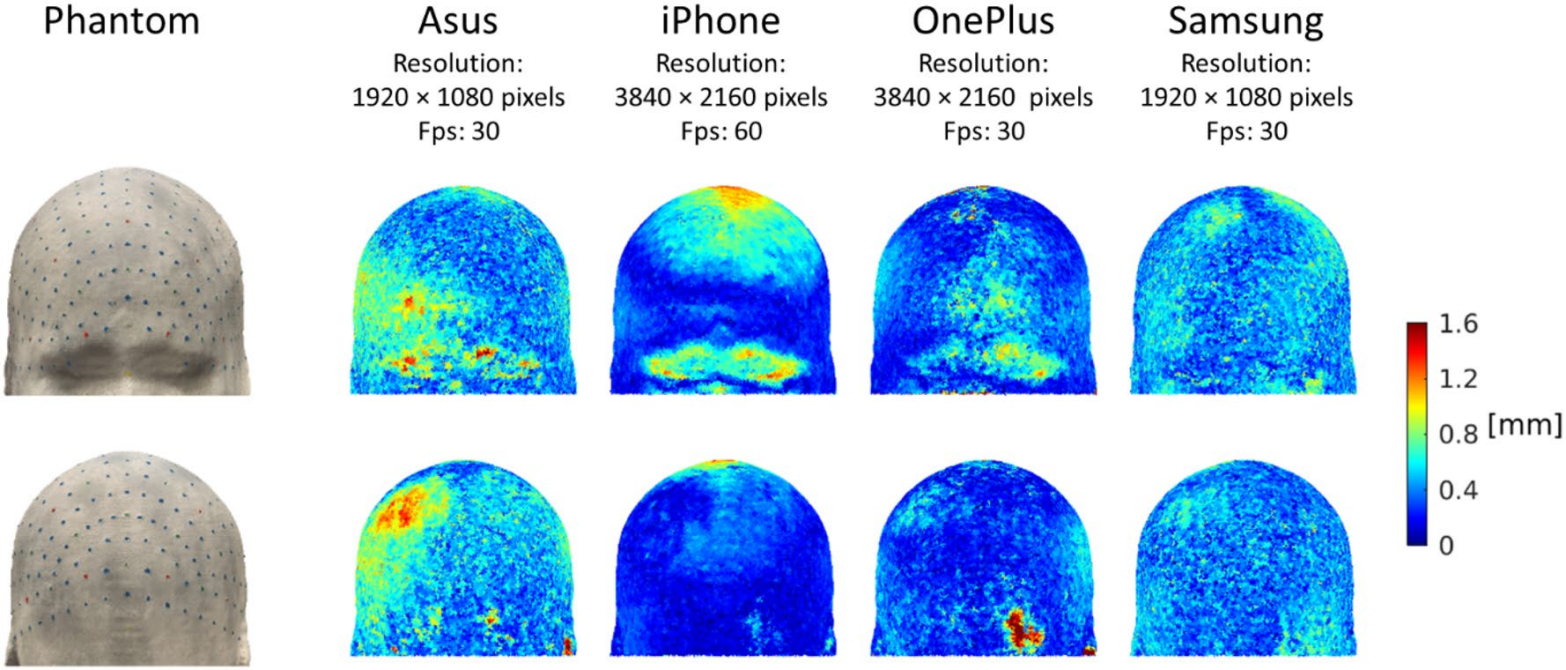
Deviation of the smartphone-derived meshes from the original phantom. Deviation values are represented with a colour code on the phantom model. Each value indicates the Euclidean distance between a node on the original phantom and the closest node on the reconstructed model.

Figure 3 displays the accuracy of the reconstructed model. While both Asus, iPhone and OnePlus displayed some localized regions with decreased accuracy, Samsung showed a more homogeneous distribution of errors across the head. On average, the most accurate model was the iPhone-derived mesh, the smartphone with highest resolution and fps (median deviation: 0.2 mm, median absolute deviation (MAD): 0.1 mm), whereas the biggest difference was obtained with the Asus-derived mesh (median deviation: 0.5 mm, MAD: 0.1 mm).

**Figure 4:**
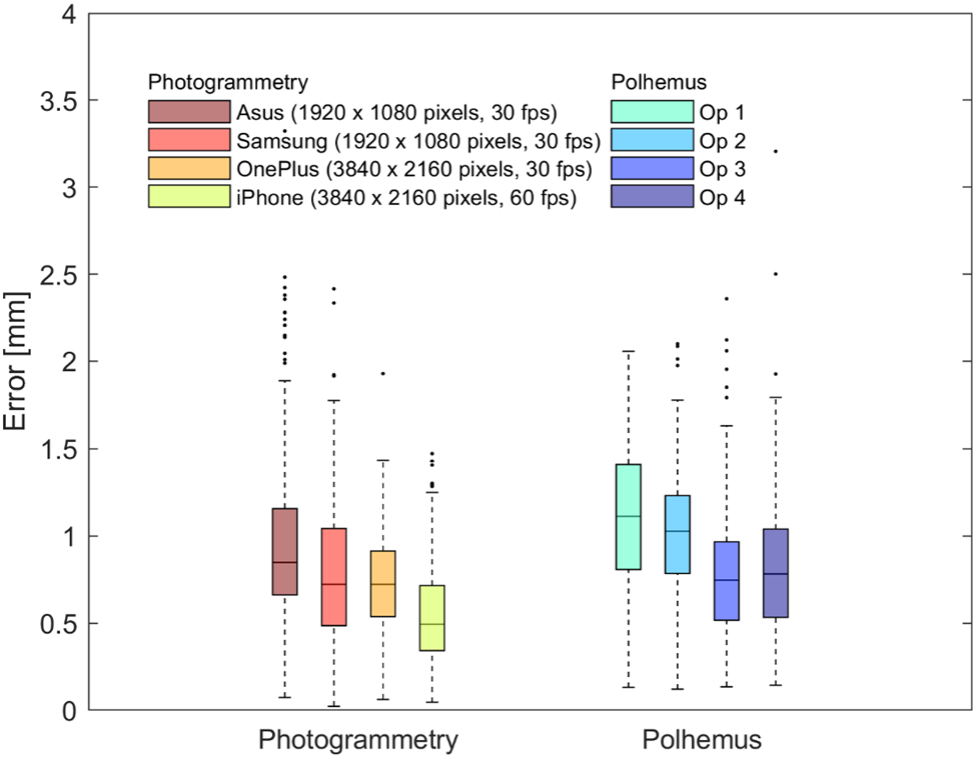
Localization errors for the sensor positions obtained with photogrammetry and Polhemus for the head phantom. Boxplots represent the distribution of the errors across sensor positions, separately for each employed smartphone for the photogrammetry-based method (on the left) and for each operator for the Polhemus (on the right). Bottom and top edges of the box indicate the 25th and 75th percentile, whereas the central mark indicates the median. Outliers are depicted as dots.

The median (MAD) localization errors for Polhemus and photogrammetry-based method across all sensors, cameras and acquisitions were, respectively, 0.9 (0.3) mm and 0.7 (0.2) mm. Figure 4 shows the distribution of errors across the sensors for the two techniques. The most accurate sensor positions were obtained with the combined use of the photogrammetry-based method and the smartphone with highest resolution and fps, the iPhone Xs (median: 0.5 mm, MAD: 0.2 mm).

### Registration study

**Figure 5:**
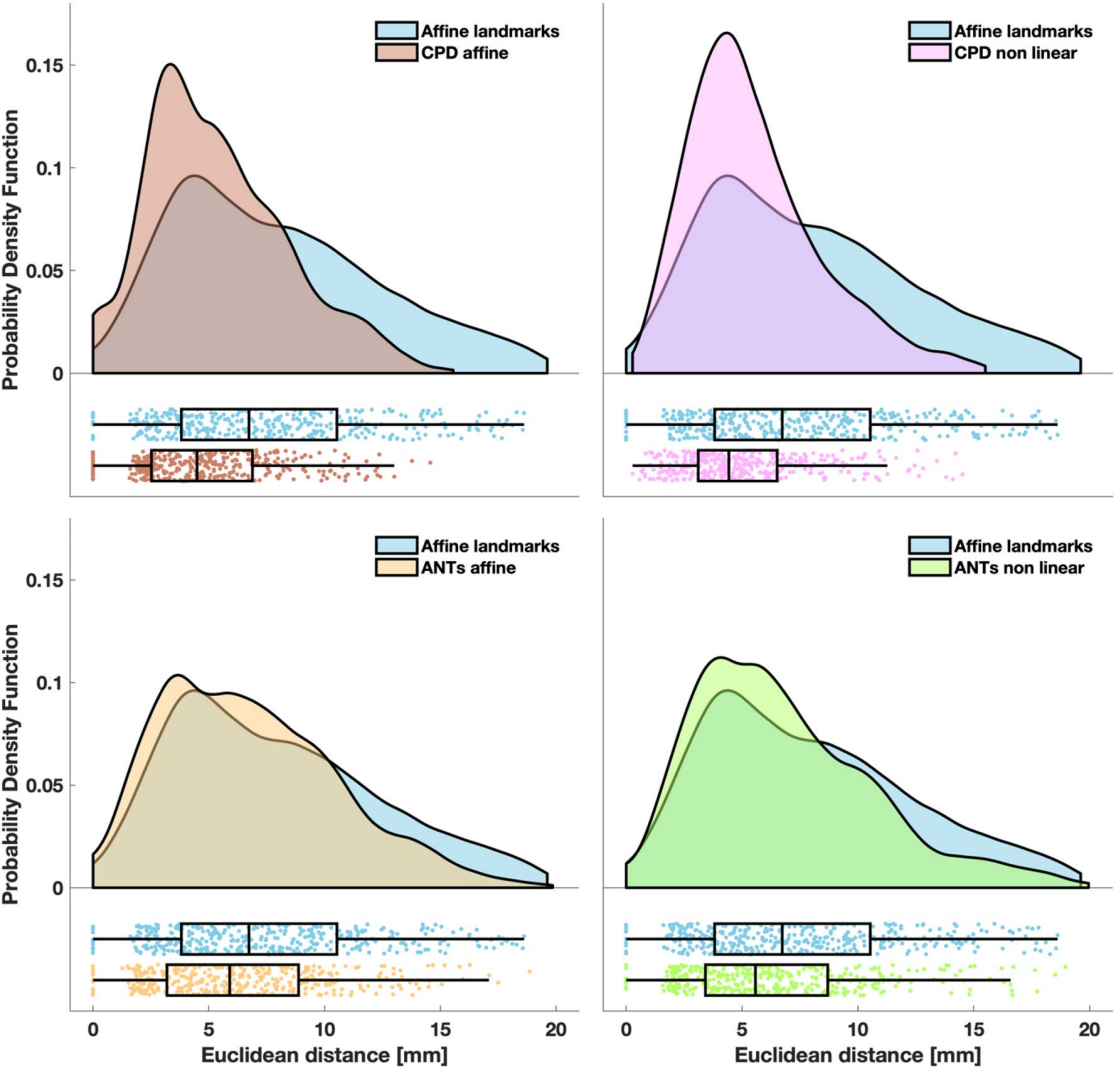
Euclidean distance between the benchmarks of the sensor positions and the registered positions. The probability density function of the localization errors obtained with CPD affine (top left panel), CPD non linear (top right panel), ANTs affine (bottom left panel) and ANTs non linear (bottom right panel) are superimposed to the probability density function of the localization errors obtained with the affine transformation based on landmarks. In the lower part of each panel, the same data are presented as a box plot. Left and right edges of the box indicate the 25th and 75th percentile, whereas the central mark indicates the median. The whiskers extend to the most extreme data value not considered an outlier. Each dot represents a sensor of a participant. Probability density functions were estimated with the kernel density estimation method and graphs were generated as described in Allen et al.^65^.

**Figure 6:**
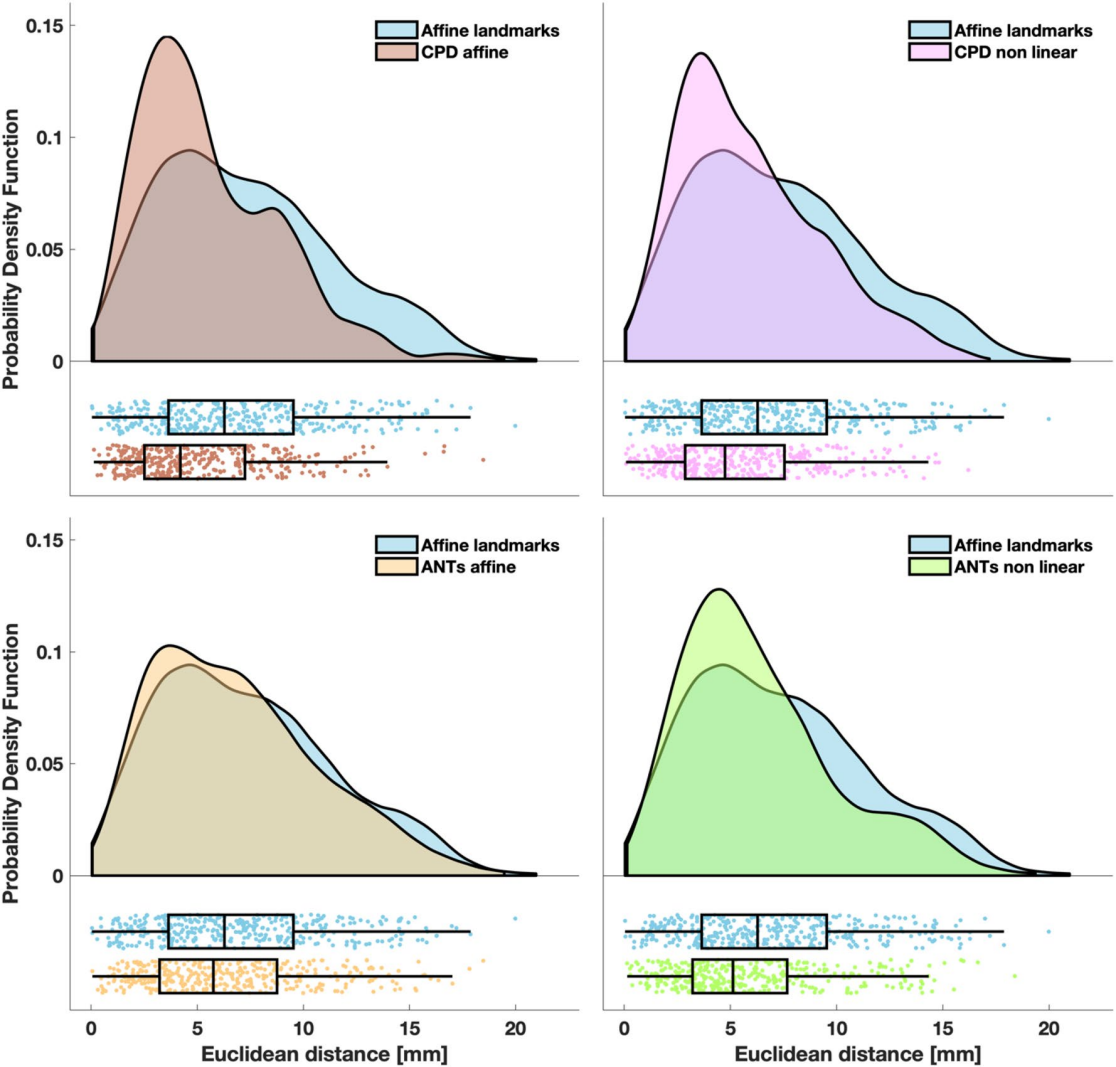
Euclidean distance between the benchmarks of the location of the peak of the fluence distribution and the location of the peak of the registered fluence distributions. The probability density function of the localization errors obtained with CPD affine (top left panel), CPD non linear (top right panel), ANTs affine (bottom left panel) and ANTs non linear (bottom right panel) are superimposed to probability density function of the localization errors obtained with the affine transformation based on landmarks. In the lower part of each panel, the same data are presented as a box plot. Left and right edges of the box indicate the 25th and 75th percentile, whereas the central mark indicates the median. The whiskers extend to the most extreme data value not considered an outlier. Each dot represents a sensor of a participant. Probability density functions were estimated with the kernel density estimation method and graphs were generated as described in Allen et al.^65^.

Figure 5 summarizes the distribution of the Euclidean distance between the benchmarks of the sensor positions and the registered sensor positions in all participants. The same metric relative to fluence distribution is shown in Fig. 6.

The highest errors were obtained with the affine transformation based on landmarks (median Euclidean distance of 6.7 mm for sensor positions and of 6.3 mm for the fluence) whereas the lowest errors were achieved with CPD affine transformation (median Euclidean distance of 4.5 mm for sensors positions and of 4.2 mm for the fluence).

**Figure 7:**
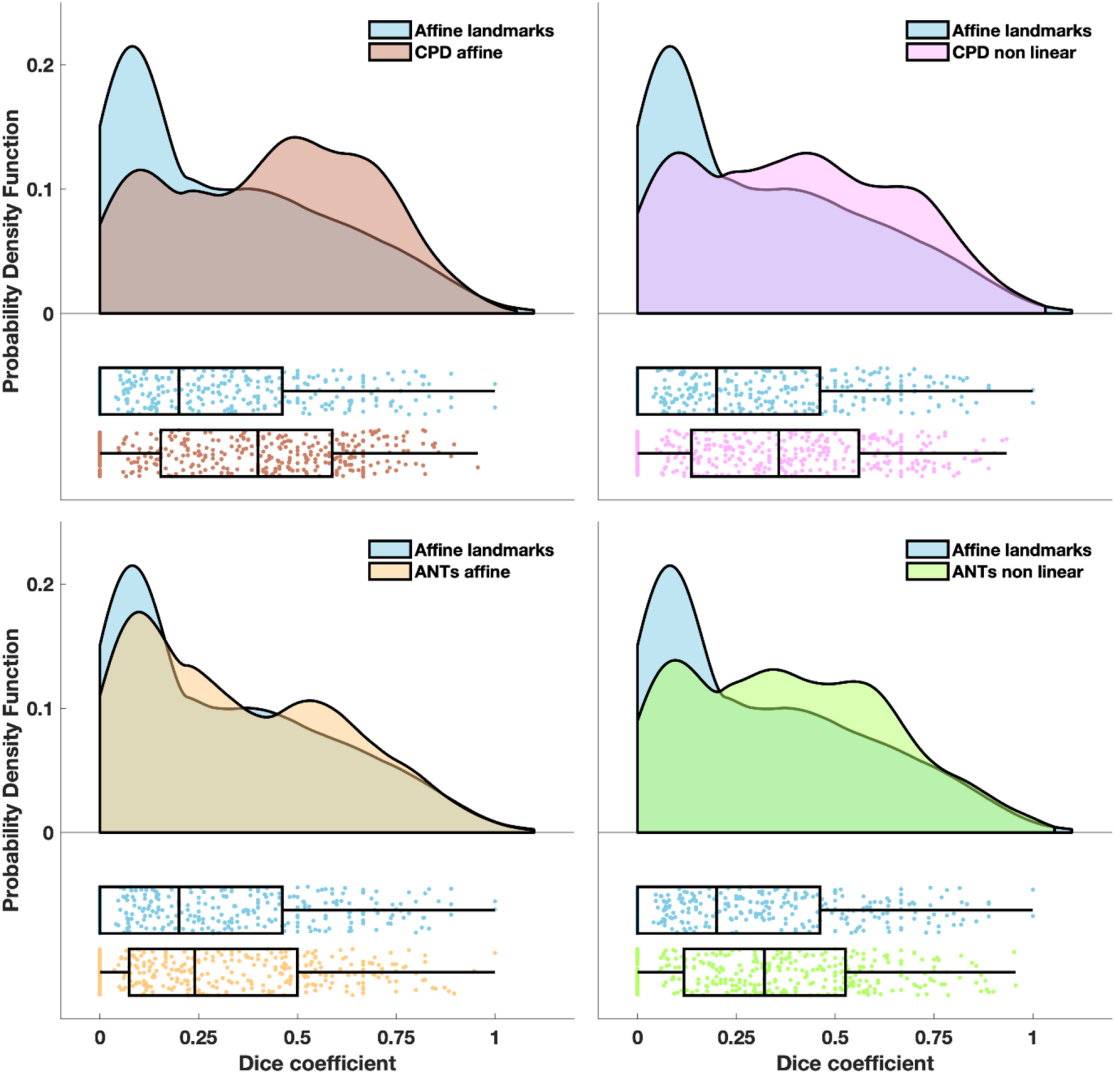
Dice coefficient between the benchmarks of the fluence distribution and registered fluence distributions. The probability density function of the Dice coefficients obtained with CPD affine (top left panel), CPD non linear (top right panel), ANTs affine (bottom left panel) and ANTs non linear (bottom right panel) are superimposed to probability density function of the localization errors obtained with the affine transformation based on landmarks. In the lower part of each panel, the same data are presented as a box plot. Left and right edges of the box indicate the 25th and 75th percentile, whereas the central mark indicates the median. The whiskers extend to the most extreme data value not considered an outlier. Each dot represents a sensor of a participant. Probability density functions were estimated with the kernel density estimation method and graphs were generated as described in Allen et al.^65^.

Figure 7 reports the distribution of the Dice coefficient between the benchmarks of the fluence distribution and the transformed fluence distribution in all participants. The smallest overlap was obtained with the affine transformation based on landmarks (median Dice coefficient of 0.2), whereas the highest overlap was achieved with CPD affine transformation (median Dice coefficient of 0.4).

With all metrics, the errors/distortions obtained with the affine transformation based on landmarks were significantly higher (max *p* = 0.02, min *z* = 2.24) than the errors/distortions obtained with the CPD/ANTs -based approaches. The errors/distortions obtained with the CPD affine approach were significantly lower (max *p* = 0.04, min *z* = 2.02) than the errors/distortions obtained with the other registration methods, but for the CPD non linear one when considering Euclidean distance relative to sensor positions. In this case, no statistically significant difference was found.

If we compute the average performance improvement across subjects for each registration method compared to the affine transformation based on landmarks, regardless the metric, the approach yielding the best improvement is CPD affine, both considering the sensor position and the fluence distribution (mean Euclidean distance decrease: 31% for sensor position, 25% for fluence distribution, mean Dice coefficient increase: 118%). The approach yielding the least improvement relative to sensor positions and fluence distribution was ANTs affine (mean Euclidean distance decrease: 12% for sensor position and 8% for fluence distribution, mean Dice coefficient increase: 36%).

The computational time of the different registration approaches are listed in Table 1.

**Table 1:** Mean computational time required by the different registration approaches.

Registration approach	Computational time Mean (SD)
Affine landmarks	0.083 (0.003) s
CDP affine	10.9 (0.1) min
CPD non linear	81.5 (5.7) min
ANTs affine	166.1 (14.3) min
ANTs non linear	185.2 (10.2) min

## Discussion

The first aim of the study was to validate the photogrammetry-based method and to compare its performance with the gold standard technique based on the electromagnetic digitizer. To carry out this step, we detected the positions of the 10-5 system in a head phantom with both the electromagnetic digitizer by four operators and the photogrammetry-based method with four different smartphones. Performances were evaluated based on the Euclidean distance between the estimated position and the ground truth position. We also evaluated the accuracy of the reconstructed models when different smartphones were used. The most accurate models were obtained with the smartphones with the highest resolution, which provided the lowest localization error of the sensor positions, whereas the lowest accurate ones, associated to the highest errors in sensor localization, were obtained with the smartphones with lower resolution (Figs. 3 and 4). It should be noted, however, that all smartphone models provided accurate results, with errors lower than usual sensor size (~ 1 cm).

Note that the accuracy of electromagnetic digitization and photogrammetry also relies on the registration step required to map the detected positions to the same reference system of the head phantom. Therefore, the error can be considered the sum of the registration error and the method-dependent localization error. Since the registration step was carried out in the same way for both techniques, we assumed that the registration error equally affected the results.

Both techniques were able to accurately detect sensor positions (errors lower than 1.5 mm on average), with a slightly lower error obtained with the photogrammetry-based approach. While the variability in the error distribution depends on the resolution and quality of the lenses of the smartphone for the photogrammetry-based method, the variability in the error distribution for Polhemus only depends on the operator manual ability, thus introducing a subjective error when using Polhemus. The median localization error obtained with the photogrammetry-based method (0.7 mm) is in line with other photogrammetry studies^24–26,30^ which reported mean errors ranging between 0.41 and 1.3 mm and 3D scanning studies^31,32,62^ which reported mean errors ranging between 0.9 and 1.5 mm. The median localization errors obtained with Polhemus (0.9 mm) is in line with that reported in Russell et al.^22^ and slightly lower than that reported in Taberna et al.^32^. Baysal and Sengul^24^, Dalal et al.^62^ and Clausner et al.^26^ found, instead, that the localization error associated with the use of the electromagnetic digitizer was around 7 mm. This discrepancy with our results can likely be explained by methodological differences relating to different sizes and shape of target sensors. In the above cited studies, the electromagnetic digitizer was used to localize EEG electrodes, whose area is at least ten times bigger than the area of the markers we digitized. It is extremely difficult for a user to place the stylus pen exactly at the centre of the electrode, thus introducing a subjective error. It is likely that the small dimension and the hollow shape of the markers on our head phantom may have ease the user’s task by driving the stylus exactly where it was expected to be and therefore lowered their error. The performance of the photogrammetry-based method, instead, does not depend on the dimension of the marker since the centre is computed as the centre of mass of the selected points. The accuracy of photogrammetry-based approaches could suffer when dealing with high-density montages with sensors very close to each other. In this case, two or more sensors could be detected as a single cluster. A possible solution, which should avoid loss in accuracy, could be to glue a small coloured circular sticker in the center of the sensor as marker of sensor position, thus increasing the distance between close sensors.

The photogrammetry-based method allows the experimenter to save, on average, a third of the time when a high-density marker configuration is employed. The time required to record sensor positions with the electromagnetic digitizer increases linearly with the number of sensors, whereas the time required by the photogrammetry-based approach is constant.

Since our photogrammetry-based method was tested with a video recorded while the phantom was being turned, we hypothesize the proposed method does not require the participant to stay motionless. A further study should be conducted to infer whether sudden movements during the video recording could affect the mesh generation or whether it is sufficient to manually remove the resulting blurred images before reconstructing the point cloud, as shown in Barbero-Garcìa et al.^63^. There are situations (e.g., with infants) where the total experimental time, as well as the compliance of the subject, is very limited. In these cases, the photogrammetry-based approach could save researchers’ time and effort and reduce errors in detecting sensor positions.

It is important to highlight that the time saved by researchers during the acquisition of the sensor positions with photogrammetry-based method comes at the expenses of a longer post-processing time compared to techniques based on electromagnetic digitizers. After data collection, the photogrammetry-based method requires additional time to process the video and obtain the head mesh from which the markers can be automatically detected. It should be noted, however, that this post-processing time, if the process is completely automatic, does not occupy researchers’ time. A study by Barbero-Garcìa et al.^63^, for example, presented an automatic solution for the creation of a 3D head model starting from the acquisition of multiple frames with a smartphone.

Since the aim of the validation study was to infer whether a smartphone could be reliably used to localize EEG/fNIRS sensors with high precision, the mesh was created with the original video resolution. This process required, on average, about 2 h. A decrease in the resolution employed to build the mesh should significantly decrease the post-processing time without necessarily reducing accuracy. The resolution of the smartphones Samsung and Asus, indeed, was half the resolution of the smartphones OnePlus and iPhone, but their error distributions were not worse than those obtained with the Polhemus (Fig. 4). This suggests that 1920 × 1080 can be considered as an upper bound for the required image resolution and that therefore the mesh generation does not require more than 65 min on a typical laptop. Another aspect to consider is that the markers on our head phantom had a diameter of 3 mm, whereas common dimensions for EEG and fNIRS sensors are around 1 cm. We expect the reconstruction of larger objects to require both a lower number of frames and a lower resolution, further reducing the computational post-processing time for generating the mesh. Further studies should be conducted to define the minimum number of frames to be used and the maximum down-sampling factor applicable to the images based on the size of the details to be reconstructed.

The proposed photogrammetry-based method is also more cost-effective than other approaches since it is significantly less expensive than the other photogrammetry/3D scanning methods as well as the electromagnetic digitizer. Furthermore, from the smartphone display the operator can always see what is being recording. This aspect should not be underestimated since with the 3D scanning methods this is not possible: the information provided by these methods during the acquisition does not allow the experimenter to predict the quality of the 3D model that will be generated.

Eventually, a note on the error that users could introduce when using the electromagnetic digitizers compared to the photogrammetry-based approach. The accuracy in detecting the sensor position with electromagnetic digitizers relies on the user’s ability to point the stylus exactly at the centre of the sensor. If the stylus is not correctly located, this could introduce an error in the sensor position. If this error occurs on one of the landmarks’ positions, the registration of all sensor positions will be affected by this error.

The second aim of the study was to test whether the individual head surface acquired with photogrammetry can be a valid aid to improve the registration of the individual sensor positions to the MRI atlas. To investigate this aspect, we compared the standard technique (affine transformation between cranial landmarks) to four different registration approaches. To the best of our knowledge, this is the first attempt to directly map the individual surface acquired with photogrammetry/3D scanning to a template surface, without the individual MRI scan as an intermediate step.

Although the errors of all registration approaches were comparable in their order of magnitude, a significant reduction in all metrics was consistently obtained when using the surface information in the registration compared to when using the cranial landmarks alone. The most accurate registration approach consisted in a rigid transformation using nasion, Cz, left and right preauricular points followed by an affine transformation computed with the CPD algorithm^44^ between the two surfaces. Since the aim of the rigid transformation is to provide only a rough alignment between the two surfaces, small errors in cranial landmarks identification would not affect the accuracy of the final registration result. It is worth highlighting that the landmarks, on which this rigid transformation is based, do not include inion. This is an important aspect, since the inion is the most difficult cranial landmark to identify, both on a real and virtual head.

The CPD non linear approach yielded results comparable with the CPD affine approach, whereas the two approaches based on a volume transformation performed worse than CPD, showing however improved results compared with the affine transformation based on landmarks. The two head surfaces to be aligned are quite similar in their macro-features, therefore, non linear registrations might not be required. Non linear registrations are also less reliable than linear registrations, since they possess more degrees of freedom. In this context, an affine transformation seems to be the ideal compromise. Volume-based registrations should provide comparable results to CPD registrations since they use the head surface information. We hypothesize that this different performance might be due to the steps transforming the surface to a volume (e.g., the choice of the grid step resolution) and then mapping back the positions obtained in the volume to the surface, which might introduce additional displacement errors.

Our results indicate that the registration approach based on CPD affine should yield a more accurate EEG source localization, since 5.0 mm has been shown to be the maximum accepted error on sensor locations to obtain negligible errors on source localization^64^. The median values of the Euclidean distance relative to sensor positions across sensors and subjects was 4.5 (MAD = 2.1) mm with CPD affine, whereas for the affine transformation based on landmarks was 6.7 (MAD = 3.1) mm. We recommend using a photogrammetry-based approach with CPD affine registration in studies requiring accurate sensor localization (e.g., source reconstruction or image reconstruction studies), whereas the easier affine transformation based on landmarks could be a valid option in studies not requiring high accuracy in sensor localization.

One drawback of the CPD affine approach compared to the affine transformation based on landmarks is the computational time, which was on average around 11 min for the former compared to less than 1 s for the latter. We expect this computational time to be reduced by increasing the down-sampling factor of the surfaces, but further studies are required to investigate how decreasing the number of mesh points affects the registration error. We believe that, except for situations requiring real-time application of the registration approach, the computational time of CPD would not have a huge impact on the analysis pipeline of both EEG/fNIRS users.

In this study we tested the localization and registration errors in an ideal situation, to evaluate the real impact of the detection techniques and registration approaches. However, both EEG and fNIRS systems are made with bundles of fibres/cables and bulky sensors located on the participants’ head. Due to the presence of the cables, head surfaces derived with the photogrammetry-based approach will be larger than the actual head size, thus affecting the results of the registration. Furthermore, sensor locations based on the position of the upper surface will be a few millimetres to one centimetre above the scalp. A further step will be therefore required, that is to project the detected sensor locations to the head surface. We envisage an easy and straightforward possible solution to this problem, that is to record two videos of the subject’s head, one with the cap without the sensors and the other one while the subject is wearing the cap with the sensors attached. The former video could be used to obtain a realistic surface of the participant’s head, which can be used to estimate, with CPD, an accurate affine transformation between the participant’s surface and the atlas. The latter can be used to identify sensor positions, which can be then orthogonally projected on the participant’s head surface. An accurate registration between the two meshes can be obtained exploiting nose and facial features. Future studies should investigate the feasibility of this solution and the additional localization errors of this further step. Using the photogrammetry-based method might be problematic when dealing with infants because it is not possible to record a single video of their whole head whilst held on the mother’s lap. This problem could be solved either by recording multiple videos whilst the infant is held in different positions and then merging all frames before deriving the mesh, or by deriving the meshes of the visible portions of the head from different videos and merging the surfaces with software like Meshlab^50^.

In conclusion, in this paper, we described and validated a low-cost photogrammetry-based approach that can be easily employed to identify sensor locations by recording a video of the subject’s head with a smartphone. Furthermore, we compared four different registration approaches that exploited the information of the subject’s head shape measured with photogrammetry with the gold standard registration approach based on an affine transformation between landmarks. Our results highlighted that the most accurate approach to register individual sensor positions to an atlas is the combination of the photogrammetry-based technique with an affine transformation between the individual and atlas head surface performed with CPD.

## Data Availability

The data of the validation study, results of both validation and registration study and all the code developed in this paper have been released via https://github.com/sbrigadoi/Smartphone-Photogrammetry.
