# Author Correction: Smartphone-based photogrammetry provides improved localization and registration of scalp-mounted neuroimaging sensors

**DOI:** 10.1038/s41598-023-36671-7

**Published:** 2023-06-14

**Authors:** Ilaria Mazzonetto, Marco Castellaro, Robert J. Cooper, Sabrina Brigadoi

**Affiliations:** 1 Department of Developmental and Social Psychology, University of Padova, via Venezia 8, 35131 Padua, Italy; 2 Department of Information Engineering, University of Padova, via Gradenigo 6/b, 35131 Padua, Italy; 3 Department of Neuroscience, Biomedicine and Movements, University of Verona, P.le L.A. Scuro 3/A, Verona, Italy; 4 DOT‑HUB, Biomedical Optics Research Laboratory, Department of Medical Physics and Biomedical Engineering, University College London, London, WC1E 6BT UK

Correction to: *Scientific Reports* 10.1038/s41598-022-14458-6, published online 27 June 2022

The original version of this Article contained an error in Reference 27, which was incorrectly given as:

Jaffe-Dux, S., Bermano, A. H., Erel, Y. & Emberson, L. L. Video-based motion-resilient reconstruction of three-dimensional position for functional near-infrared spectroscopy and electroencephalography head mounted probes. *Neurophotonics* 7, 1 (2020).

The correct reference is listed below:

Jaffe-Dax, S., Bermano, A. H., Erel, Y. & Emberson, L. L. Video-based motion-resilient reconstruction of three-dimensional position for functional near-infrared spectroscopy and electroencephalography head mounted probes. *Neurophotonics* 7, 3, 035001-035001 (2020).

The original Article has been corrected.

